# Supplementary material for: Mechanisms governing the pioneering and redistribution capabilities of the non-classical pioneer PU.1
Source: Nat Commun. 2020 Jan 21;11:402. doi: 10.1038/s41467-019-13960-2 (PMC6972792; doi:10.1038/s41467-019-13960-2)
Supplement: Supplementary file 7 — Source data [file 41467_2019_13960_MOESM7_ESM.zip › Source_Data/FigureS2/FigureS2B_MotifScanOutput/KmeansCluster10/homerResults/motif25.similar.html]

motif25

## Information for motif25

T
G
C
A
T
G
A
C
T
G
A
C
G
C
T
A
T
G
A
C
G
C
T
A
C
A
G
T
G
A
T
C
T
C
G
A
T
G
C
A
C
T
G
A
A
C
T
G
  
Reverse Opposite:  

T
G
A
C
G
A
C
T
A
C
G
T
A
G
C
T
C
T
A
G
G
T
C
A
C
G
A
T
A
C
T
G
C
G
A
T
A
C
T
G
A
C
T
G
A
C
G
T
  

|  |  |
| --- | --- |
| p-value: | 1e-25 |
| log p-value: | -5.940e+01 |
| Information Content per bp: | 1.708 |
| Number of Target Sequences with motif | 111.0 |
| Percentage of Target Sequences with motif | 2.99% |
| Number of Background Sequences with motif | 360.4 |
| Percentage of Background Sequences with motif | 0.82% |
| Average Position of motif in Targets | 95.3 +/- 54.9bp |
| Average Position of motif in Background | 99.6 +/- 66.7bp |
| Strand Bias (log2 ratio + to - strand density) | -0.2 |
| Multiplicity (# of sites on avg that occur together) | 1.00 |
| Motif File: | file (matrix) reverse opposite |

### Similar de novo motifs found

|  |  |  |  |  |  |  |  |
| --- | --- | --- | --- | --- | --- | --- | --- |
| Rank | Match Score | Redundant Motif | P-value | log P-value | % of Targets | % of Background | Motif file |
| 1 | 0.837 | T C G A G T A C A T G C C G T A T G A C C G T A G A C T G A T C G T C A G C T A | 1e-21 | -49.681114 | 1.67% | 0.31% | motif file (matrix) |
| 2 | 0.955 | A G T C A G C T A G C T C G A T A C T G G C T A C G A T A C T G C A G T C T A G C T A G | 1e-20 | -46.897333 | 4.85% | 2.12% | motif file (matrix) |
| 3 | 0.811 | T C G A T A G C G C T A G A C T G T A C C T G A G T C A G C T A T A C G T G A C | 1e-17 | -40.325621 | 7.46% | 4.16% | motif file (matrix) |
| 4 | 0.919 | C G T A T C G A A G T C G T A C C G T A G T A C C G T A G C A T G A T C G T C A T G C A C G T A A T C G T G A C | 1e-17 | -39.887579 | 0.67% | 0.04% | motif file (matrix) |
